# CFDMI-SEC: An optimal model for copy-move forgery detection of medical image using SIFT, EOM and CHM

**DOI:** 10.1371/journal.pone.0303332

**Published:** 2024-07-23

**Authors:** Ehsan Amiri, Ahmad Mosallanejad, Amir Sheikhahmadi

**Affiliations:** 1 Department of Computer Engineering, Sanandaj Branch, Islamic Azad University, Sanandaj, Iran; 2 Department of Computer Engineering, Sepidan Branch, Islamic Azad University, Sepidan, Iran; Banca d’Italia, ITALY

## Abstract

Image forgery is one of the issues that can create challenges for law enforcement. Digital devices can easily Copy-move images, forging medical photos. In the insurance industry, forensics, and sports, image forgery has become very common and has created problems. Copy-Move Forgery in Medical Images (CMFMI) has led to abuses in areas where access to advanced medical devices is unavailable. The proposed model (SEC) is a three-part model based on an evolutionary algorithm that can detect fake blocks well. In the first part, suspicious points are discovered with the help of the SIFT algorithm. In the second part, suspicious blocks are found using the equilibrium optimization algorithm. Finally, color histogram Matching (CHM) matches questionable points and blocks. The proposed method (SEC) was evaluated based on accuracy, recall, and F1 criteria, and 100, 97.00, and 98.47% were obtained for the fake medical images, respectively. Experimental results show robustness against different transformation and post-processing operations on medical images.

## 1. Introduction

Intentional manipulation of an image to change its information is called image forgery [1, 2]. The most critical forgeries are adding, deleting, or identifying objects in the image. Changing any feature or content of the image will result in forgery if it leaves no trace of the change in the result [3]. The number of software that edit the image free is enormous. Therefore, image forgery is widespread. In contrast to image forgery, image forgery detection algorithms must be strong enough to detect image forgery [3, 4]. Object tracking [5], matching [6], feature selection, pre-processing, and image super-resolution reconstruction algorithms [7, 8] can be considered in forgery detection.

Copy-move forgery (CMF) [9, 10] or simulation fraud is one of the most common types of image forgery. In the copy-move image forgery, the part of the image with the appropriate feature is copied and then pasted by selecting the proper location [11]. The primary purpose of forging copy-move is to hide objects and image aspects.

**Fig 1:**
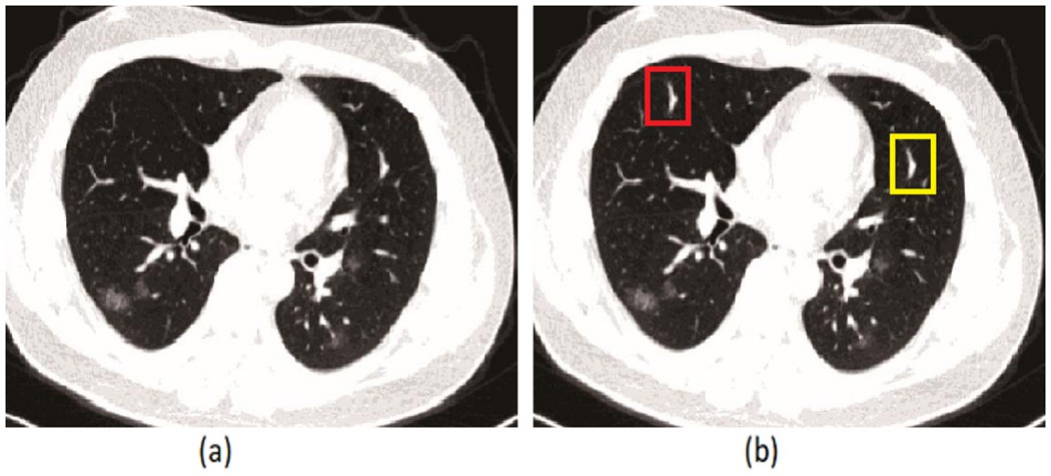
An example of image forgery [12]. (a) The first image is the original image captured by a camera, and no changes have been made. (b) The second image has been edited. A yellow and red box can be seen in the image. The red box was the best place to put the yellow box.

The same areas in the copy-move forgery can have different sizes and shapes and can be the forged part of the image one or more times in different places (Fig 1) [12]. Newly, various methods have been introduced in copy-move forgery detection. PCET [13] and AKAZE [14] methods are among these. The authors applied deep learning in 2020 to 2024 [15–17].

Forgery in medical images [18] is one of the most critical issues in forensic medicine. People get insurance benefits by forging medical pictures. It has sometimes been observed that an athlete refuses to perform exercises or competitions by changing medical images [19]. By falsifying their medical images, workers persuade employers to grant unique benefits. If applied to a medical image, copy-move forgery misleads the physician or hospital staff [20]. In copy-move forgery, a part of the body is placed in the same part and confuses the doctor [21].

The motivation for Copy-Move Forgery Detection of Medical Images (CMFDMI) is to detect manipulated images. Image forgery detection is critical, and researchers are focused on CMFD and have achieved excellent results. According to the studies, copy-move forgery can be classified into two general methods [20] based on block and key-point. Suganya et al. introduce a key-point (KP) forgery detection method based on golden ball-based optimization to get critical features faster [18]. This article has analyzed 300 medical images. Despite the improvements in feature selection, it still cannot overcome the challenges of keypoint-based methods. Improving the behavior of this algorithm can be done by combining block methods. It seems that selecting fake blocks and expanding the affected area with the help of critical features is a suitable method for detecting fakes in medical images.

In block-based image forgery detection methods, the image is divided into several blocks, and the main features are obtained according to the selected blocks [13, 22]. Several different properties are selected from blocks in a block-based method. For instance, the principal component analysis (PCA) method was introduced in [23]. The PCA method is used to describe blocks of low complexity. Local Binary Pattern (LBP) [24] and Discrete Cosine Transform (DCT) [25] are other methods.

Keypoints are extracted from the image in methods that use a keypoint. Keypoint-based detection techniques play a crucial role in revealing copy-move evidence because of their resilience to extensive geometric transformations [26]. The most crucial method among key-point methods is the scale variable property conversion (SIFT) method [27], which many studies use as a suitable descriptive method for detecting forgery. Amerini, in 2011, detected copy-move forgery based on the SIFT feature, which has obtained excellent results [28, 29]. The SIFT method has been modified and improved in many studies [30].

In the last few years, methods have been introduced for placement in paintings, which can be suitable methods for creating clever forgeries in the future. Deep neural network methods such as generative adversarial attacks [31] and Large-Scale JPEG Image Steganalysis [32] and combining features with the help of Illumination Color Classification [33] have been able to obtain good results. To detect such forgeries, intelligent models based on evolutionary algorithms should be introduced so that they can better search for prominent points. One of the recently considered methods is evolutionary methods in optimizing the detection answers of fake copy-move images.

**Fig 2:**
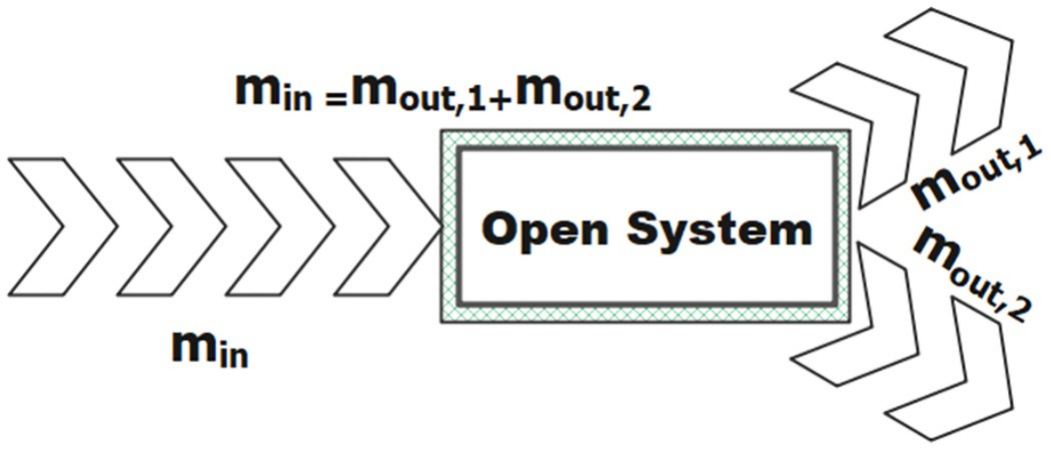
Input and output in the mass balance equation [38]. The input energy is equal to the sum of the output energies, provided that energy storage or loss is not considered.

So far, many evolutionary algorithms [34–37] have been introduced. Evolutionary algorithms such as Equilibrium Optimization (EO) can solve various problems based on intelligent principles [38]. The mass balance equation is obtained according to the amount of mass entered into the system. The mass balance equation for the input is equal to the sum of the first output mass and the second output mass (Fig 2).

Sometimes, it occurs in the accumulation system, which must maintain the stable energy equation and the state of general equilibrium. In the case of accumulation, the sides of the equation must be equal [38, 39].


$$V\frac{dC}{dt}=QC_{eq}-QC+G$$ (1)

According to Eq 1, the mass production rate equals the number of changes in the input per second. In Eq 1, C is mass per cubic meter, Q is the velocity, V is volume, and dc/dt indicates the volume change rate [39].

According to these cases, Q * C will be the system’s input, and its unit is in kilograms in seconds. QC is also the concentration that goes out of control volume [38].

Eq 1 is a first-order differential equation showing the general mass equilibrium equation. In Eq 1, the mass change over time equals the amount of mass entering [40].

If there is no change in the system and Vdc / dt is zero, a steady state of equilibrium is achieved. A stable equilibrium is a state in which a change in an equation does not occur during the period of stability. Therefore, the parameters of the stable equation do not change over time. Generally, a constant equilibrium state is obtained when the input and output of the equation are constant [38]. The equilibrium optimization algorithm is a numerical method used to solve equilibrium optimization problems. These algorithms are generally used to find equilibrium points in equilibrium optimization problems, so that there is a balance between all variables and constraints. This algorithm seeks to find the optimal equilibrium points of various problems by using a combination of equilibrium and optimization methods. Applying the equilibrium optimization algorithm to address image processing issues presents numerous challenges. It will be challenging to determine the correct initial equilibrium points, how to achieve equilibrium, and alter concentrations when the parameters are image and image blocks.

For this paper, the main contributions are as follows:

- Copy-Move forgery is investigated and detected fraud in medical images.
- Using an optimization method, fake blocks are optimally detected, increasing the accuracy of detecting fake obstructions in medical images.
- With the help of the histogram matching method, affected areas are expanded.

With the help of an evolutionary algorithm, this paper introduces an optimal method for detecting forgery in an image. Section 2 presents a copy-move forgery detection algorithm based on a three-stage keypoint model, optimization, and similarity algorithm called SEC. Section 3 presents the experiments, and Section 4 presents the Conclusion.

## 2. Related works

Copy-move forgery is a common type of image forgery [2]. In this method, a specific part of the image is used to improve/remove features. This technique involves duplicating a part of an image and pasting it in another area of the same image to cover specific features in the real image. Various keypoint-based or block-based approaches have been developed to detect copy movement. The input image is divided into regions, or key points are extracted using feature extraction algorithms to perform detection. The proposed algorithm should be able to recognize images with rotation, scaling, and compression applied to the image.

In the block method, connected blocks of the image that are copies of each other are searched. The copied area contains a large number of overlapping blocks. Therefore, forgery is detected when there are more than a certain number of image blocks with equal distances and these blocks are connected to form two areas of the same shape. The important point in these methods is to find a suitable way to display the blocks so that the copied blocks can be recognized even if they have undergone changes. Some authors have suggested the use of different features to display blocks. In [3], the authors presented a copy-move forgery detection method against scaling operations. Runlong et al. [5] proposed a simple motion-copy forgery detection algorithm using Rauch-Tung-Striebel smoother, which achieves low false negative error and fast execution speed. Based on the method of Park et al. [6], a block face recognition scheme was introduced. Tapia et al [8] introduced the LBP search algorithm which can automatically generate the appropriate parameter value for each image. Another critical method introduced in the past by Ulutaş et al. (2013) is the extracted uniformly local binary patterns (LBPs) that were based on circular blocks [24]. The block method considered in this article is Discrete Cosine Transformation (DCT), introduced by Vega et al. in 2021 [25].

The block structure depends on the details of the image. Due to the multimodal feature in wavelet transform, several problems of signal processing and vision can be solved. Naturally, digital imaging has also been found to be effective for forensic applications. Discrete wavelet transform (DWT) is the most widely used in the detection method of region multiplication based on wavelet transform. Here, the image is decomposed into four subbands. Much work has been done on image forgery detection using discrete wavelet transform. In [10], the authors presented many motion-copy forgery detection schemes with the help of DWT. Most of the studies conducted are in the LL subbands, which is most related to the low-level features of the image. A similar approach was used in [30] using LL and HH vectors to identify repetitive regions. In [41], LL is divided into overlapping image blocks and forgery detection was performed based on the separated blocks.

Altered images are more likely to exhibit signs of malicious intent, like using pictures of minors for promoting prostitution or spreading false political claims. Following this widespread practice, various techniques have been proposed over time. Currently, neural networks like CNNs synergistically move in this direction [42]. In [43], a two-stage cascade framework was proposed for detecting small area forgeries in GAN-based medical images, such as CT-GAN. During the detection stage, the detector network was trained using small sub-images to prevent interference information in the valid regions from impacting the detector. Also, convolution networks were used to prevent overfitting of the detector. In [44], an optimal blind forgery detection using deep learning methods is introduced. Initially, the forged input images are preprocessed using an advanced histogram equalization method with a Wiener contrast filter. Effective features are extracted from clustered data using wavelet transform and VGGNet. Finally, a hybrid deep convolutional autoencoder framework is presented for optimal image forgery detection. Hosny [16] and Koul [17] introduced a deep neural network in 2022 to image forgery detection from the MICC dataset. The large quantity of images available impacts the accuracy of diagnosis using deep neural networks.

The wavelet transform has been utilized in numerous forgery detection techniques. Discrete wavelet transform faces challenges in the field of forgery detection. However, some work has been done to overcome these challenges. Kaur et al. [4] introduced a PCA model to reduce feature dimensions in forged blocks, which performed well in detecting forged regions. Zang et al. [32] introduced a forgery detection method based on JPEG images, which included a new model for detecting motion-copy forgery.

Key point methods focus on identifying and selecting areas with high entropy in the image. For each key point, a feature vector is extracted. This results in a smaller number of feature vectors, which reduces the computational complexity in feature matching and post-processing. Fewer feature vectors also lower the limit value of thresholds of post-processing operations compared to block-based methods. In Amiri et al., an optimal model of SIFT is introduced [9], and Singh et al. [30] developed a hybrid model of SIFT and SVD to withstand different types of attacks.

Two different types of feature vectors are SIFT and SURF. Kashif et al [27] used SURF point matching with multiscale analysis. In [28], Amerini et al. matched SIFT and SURF key points in their motion-copy forgery detection method. KAZE feature analysis is introduced in [14], where the scale space is constructed non-linearly. KAZE feature points and SIFT points can be well combined to detect the manipulated area more accurately. Amerini et al [29] proposed SIFT-based motion-copy forgery detection methods. In [32] presented a generalized 2NN test to localize multiple duplicated regions and cumulative hierarchical clustering to identify possible simulated region. J-Linkage algorithm [29] was introduced to improve SIFT.

Exact algorithms are able to find the optimal solution accurately, but in the case of hard optimization problems, they are not efficient enough, and their execution time increases exponentially according to the dimensions of the problems. One of the newest and most powerful evolutionary optimization methods is the equilibrium optimization algorithm, which is more capable of finding global optimal points. This algorithm simulates the behavior of equilibrium to solve optimization problems, and suitable methods for solving optimization problems are random methods [38]. However, very few examples of optimization algorithms have been used in image processing. In the study [37] in 2022, an algorithm based on the blue wall was introduced. Agarwal [34] used the emperor penguin optimization algorithm to optimize the selection of blocks. The results [25] show the superiority of the proposed method in selecting similar blocks over many block methods. In [35] used the bat optimization method, and [36] used the football game optimization method. The equilibrium optimization method is one of the newly introduced evolutionary methods, which can work on images due to its agent-oriented structure. In the study [36] in 2020, they studied on the optimization of copy-move forgery detection with the help of football game optimization algorithm. This paper proposes a new copy-move forgery detection method that considers a small number of the strongest KPs, selected from Gaussian-based KPs and FAST corner KPs. In the paper [34] published in 2021, he presented a forgery detection method based on super pixel areas. The innovation of this article is the use of the Emperor Penguin optimization algorithm in optimizing the selection areas. This paper proposes a robust motion-copy forgery detection scheme by integrating block-based and keypoint-based copy-move forgery detection techniques. The input image is first divided into non-overlapping blocks based on super pixels using a modified FCM clustering algorithm. The effects of neighboring and similar super pixels in FCM are provided by the Emperor Penguin optimization algorithm, which increases the segmentation performance.

## 3. A three-stage method for copy-move forgery detection

**Fig 3:**
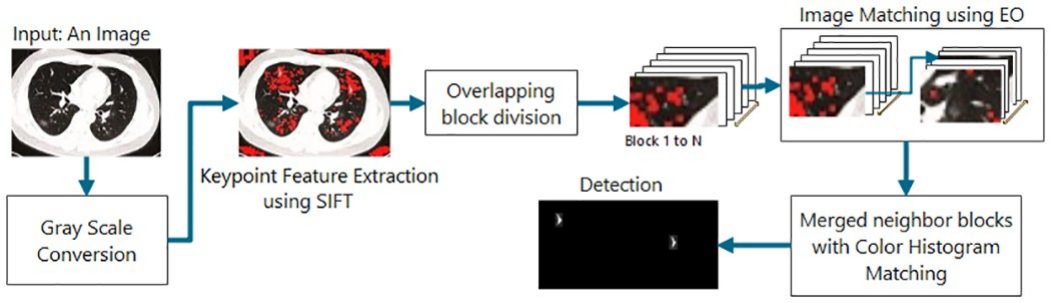
Copy-Move forgery detection using three stage model (SIFT, Equilibrium Optimization, and Histogram Matching).

This section proposes a new algorithm called SEC based on Scale Invariant Feature Transform (SIFT) [9], Equilibrium Optimization Algorithm (EOA) [38], and Color Histogram Matching (CHM) [2], as shown in Fig 3. The introduced method is a three-stage method because block methods are unable to expand the area of forgery, therefore, a model should be designed that, in addition to choosing the number of appropriate forgery blocks, can recognize the correct area of forgery.

The three-stage model can determine how much the area should grow by choosing the correct number of blocks, with the help of selected key points. The first step is to find the optimal number of blocks. The second step finds the fake points. The third step expands the fake block based on key points. In the first and second stages, forgery is detected with the help of SIFT and EOA matching. In the third stage, the results of forgery detection will have merged with Color Histogram Matching (CHM).

### 3.1. Scale-Invariant Feature Transform (SIFT)

In the first step, the proposed method extracts key points of the gray image by SIFT. Almost all digital images are RGB-based color images [9]. First, convert them into a gray scale by combining all three channels using Eq (2).


GrayImage=0.299R+0.583G+0.116B (2)

Rs, G, and B indicate the pixel intensities of red, green, and blue channels.

**Fig 4:**
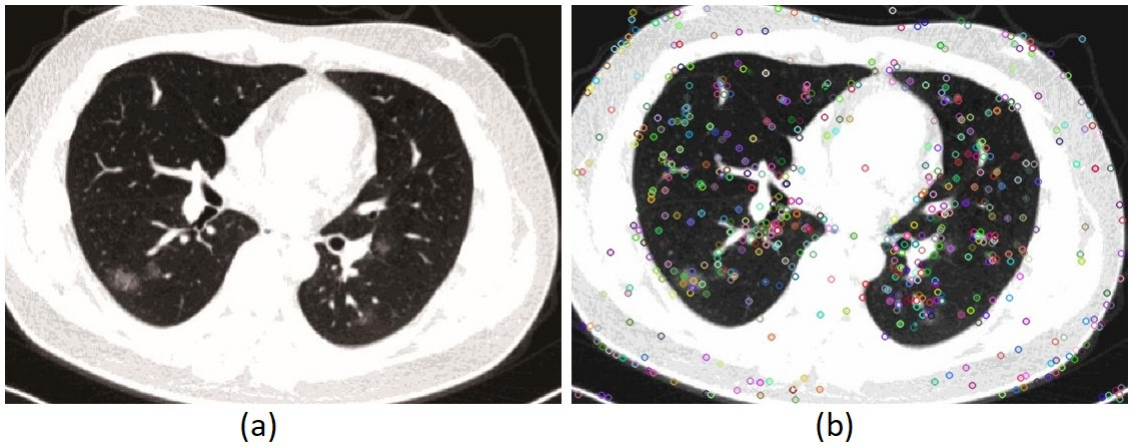
Extracted keypoints with SIFT. a) Original Image with Copy-Move forgery. b) Select point with Sift algorithm.

The color points in Fig 4 are the extracted key points. A threshold value is used in forgery detection by the SIFT method. The threshold value is obtained experimentally. The method’s practical nature makes the detection inaccurate for all images. The false matching rate increases with an increase in the threshold, reducing the precision of detected forged regions. Threshold causes some forged pixels to go undetected, leading to a poor recall rate [2].

Most of the articles that have used SIFT to detect fake points use g2NN as a similarity measure. Key points with less distance are considered similar, and false points are labeled. In the proposed model, suspicious points are discovered with SIFT, and diagnosis is made in the next step.

In Fig 4, the blue circles indicate the points identified as forgings by SIFT. In some places, forgery is not done, and the g2NN method mistakenly detects them as fake. As a result, using the SIFT and g2NN increases the false detection rate of forgery.

The points obtained in the SIFT step (Fig 4) show that parts of the image are poorly placed in the copy movement. Therefore, the need to optimize the result is felt. The next step performs this operation using the balance optimization algorithm detector.

### 3.2. Equilibrium Optimization Matching (EOM)

Block methods have higher detection power than key methods, but they are weak in expanding the area of forgery. If key points are used as suspicious points in the body of a block method, it can solve two significant problems well. After determining the suspicious points with the help of SIFT, the image is divided into several blocks of size B. The first block on the top left is selected. Subsequent blocks are created by sliding one pixel to the left, from the upper left to the lower right corner. At the end of this process, the total number of blocks for an image of size M×N must be (M-B+1) (N-B+1) [13]. If B = 8 and the image size is 512×512, then 255025 blocks of 8×8 size are obtained.

With the help of the equilibrium optimization algorithm, we will check the blocks. Initially, several blocks will be considered randomly as the initial answer. The initial solutions are measured using the equilibrium optimization algorithm, and the blocks most similar to each other are selected. The most important part of the proposed method is selecting equilibrium points and detecting forgery with the EOA evolutionary algorithm. At this stage, each block is selected in order. These blocks are entered into the Equilibrium Optimization Matching (EOM) algorithm, and the balance determination operation begins.

The equilibrium optimization algorithm is based on input and output concentration balance. The values of the answer set will also be the concentration, known by the parameter C. The initial solution is blocks of image. The blocks are obtained after applying the SIFT function. So, each block contains several key points that are considered suspicious points. Each 8x8 block has a maximum of 64 key points. Each block is a two-dimensional matrix, the concentration in the equilibrium optimization algorithm.

Other parameters of the equilibrium optimization algorithm include a1, a2, and GP, which are adjusted according to the article of Faramarzi et al. [38]. The equilibrium algorithm has other parameters set as random values in the equilibrium process. Algorithm 1 presents the complete process of detecting forgery blocks.

After the parameters are initialized, the initial population is formed based on Eq (3). Four initial concentrations are randomly selected.


$$C_{eq.pool}=\left\{C_{eq.1}.C_{eq.2}.C_{eq.3}.C_{eq.4}.C_{eq.avg}\right\}$$ (3)

The objective function is essential in the EOM algorithm and our approach. We design the objective function in this subsection, as shown in Eq (4). The value function for all available concentrations will include the average difference of the standard deviation of the current concentration with other concentrations. In the problem of forgery detection, if two blocks are similar, they will have differences close to each other. The STD is standard deviation, *Block*_*u*_ and *Block*_*i*_ are the low frequency of the current block and the other blocks, respectively.


$$Fitness\left(C_{i}\right)=\frac{\sum_{i=1}^{N}\sum {\left(STD\left({Block}_{u}\right)-STD({Block}_{i})\right)}^{2}}{N}$$ (4)

The fourth step of the proposed algorithm includes the selection of the best concentrations (Ceq. pool). The best concentrations are selected based on their order so that the first concentration in the population has a minor difference (according to Eq (4)). Other concentrations will be selected accordingly.

Each answer set (Ceq. pool) contains four and an average concentration. The average concentration is selected based on the initial proposed model of the equilibrium optimization algorithm and includes the average of four selected concentrations.

The seventh step follows the convergence of the proposed algorithm. Evolutionary algorithms follow a specific problem space in the discussion of solving benchmark problems. In the problem of forgery detection, the non-existence of the problem space and the objective function of the definition of convergence is more complicated. The first step tried to define the convergence process according to the selected blocks and the method introduced in the standard equilibrium optimization algorithm. The definitions presented in the standard equilibrium optimization algorithm were ineffective in copy-move forgery detection. Eq (3) to Eq (11) were introduced to solve the forgery detection problem.

Eqs (3) and (4) were introduced to introduce the answer population and value function. The first step in converging the equilibrium optimization algorithm is determining the λ and r, r1, and r2 random parameters with values in the [0, 1].

The update of concentrations is defined according to Eq (9), which consists of the parameters of the exponential term (F) and Generation rate (G). The exponential term (F) is calculated by Eq (5) and helps to achieve a reasonable balance between exploration and exploitation.


$$F=a_{1}sign(r-0.5)\left(e^{-\lambda STD({Block}_{i})}-a_{2}\right)$$ (5)

In Eq (5), a1 and a2 are the initial values defined, λ is a random number in [0, 1], and STD is the standard deviation.

**Algorithm 1**: Equilibrium Optimization Matching (EOM)

**Input**: Image with Copy-Move Forgery

**Output**: Copy-Move Forgery Detection

**Begin**

 I = input medical image;

 GI = gray image (I) by Eq 2;

 SIFTGI = SIFT (GI);

 BlocksGI = Set B and segmentation SIFTGI with (M-B+1) * (N-B+1);

 Select *Blocks* in output BlocksGI;

 Create C (particle as concentration) for all *Blocks*;

 **Begin** EOA algorithm

  **Step 1: Initialization.** Assign free parameter a_1_ = 2, a_2_ = 1, GP = 0.5, Select C_eq1…4_ at random.

  **Step 2: while** Iteration < Max Iteration **do**

   **Step 3:** Evaluate the fitness for each particle in *Blocks* by Eq 4.

   **Step 4:** for i = 1: N

    If (fit (C_i_) <fit (C_eq1_)) then

      Set C_eq1_ with C_i_

    Else if (fit (C_i_)>fit (C_eq1_)) & (fit (C_i_) <fit (C_eq2_)) then

      Set C_eq2_ with C_i_

    Else if (fit (C_i_)>fit (C_eq1_)) & (fit (C_i_)>fit (C_eq2_)) & (fit (C_i_) <fit (C_eq3_)) then

      Set C_eq3_ with C_i_

    else if (fit(C_i_)>fit(C_eq1_)) & (fit(C_i_)>fit(C_eq2_)) & (fit(C_i_)>fit(C_eq3_)) & (fit(C_i_)<fit(C_eq4_)) then

      Set C_eq3_ with C_i_

   End if

  End for

  **Step 5:** C_avg_ = (C_eq.1_+C_eq.2_+C_eq.3_+C_eq.4_)/4;

  **Step 6:** Construct *C*_*eq*.*pool*_ = {*C*_*eq*.1_.*C*_*eq*.*2*_.*C*_*eq*.*3*_.*C*_*eq*.*4*_.*C*_*eq*.*avg*_} (Eq 3)

  **Step 7:** for i = 1: N

     Generate random λ and r, r_1_, r2

     Construct $F=a_{1}sign(r-0.5)\left(e^{-\lambda STD({Block}_{i})}-a_{2}\right)$ (Eq 5)

     Construct $GCP=\left\{\begin{array}{cc} 0.5 & r_{1}\;r_{2}>GP\\ 0 & r_{2}<GP \end{array}\right.$ (Eq 8)

     Construct $G_{0}=GCP\;\times \left|\left({Block}_{C_{eq.1}}-\lambda \times {Block}_{i}\right)\right|$ (Eq 7)

     Construct *G* = *G*_0_ × *F* (Eq 6)

     Update Concentration $C_{i}={Block}_{C_{eq.1}}+\left({Block}_{C_{eq.1}}-{Block}_{i}\right)\times F+\frac{G}{\lambda }(1-F)$ (Eq 9)

     If *C*_*i*_ ∈ *C*_*eq*.*pool*_

      *Block*_*i*_ = *STD*(*C*_*i*_) × *Block*_*i*_ (Eq 10. **Convert to white.)**

     Else

      ${Block}_{i}=\frac{STD(C_{i})}{100}\times {Block}_{i}$ (Eq 11. **Convert to Black.)**

     End for

     Select *Block* in output function;

     Create C (particle as concentration) for all Block;

   End for

   Iteration+ = 1

  End while

 **End EOA algorithm**

**End**.

The generation rate (G) is introduced to improve the exploitation phase, which can be calculated in Eq (6).


$$G=G_{0}\times F$$ (6)

In this formula, G0 is the initial value, and F is the exponential term. Eq (5) is used to obtain F, and Eq (7) is used to obtain G0.


$$G_{0}=GCP\;\times \left|\left({Block}_{C_{eq.1}}-\lambda \times {Block}_{i}\right)\right|$$ (7)

${Block}_{C_{eq.1}}$ and *Block*_*i*_ are the first selection from the initial population set and the current concentration, respectively. In Eq (7), the value obtained from GCP is made by Eq (8). It is defined as the production rate control parameter, which includes the possibility of participation of the production term in the updating process. In Eq (8), r1 and r2 are random numbers in [0, 1].


$$GCP=\left\{\begin{array}{ll} 0.5r_{1} & r_{2}>GP\\ 0 & r_{2}<GP \end{array}\right.$$ (8)

The update of the concentrations completes the convergence process. Convergence means the change of blocks, and finally, the image reaches a balance in two forgery and non-forgery parts. The update of concentrations is done by Eq (9). This equation adjusts the values of blocks based on F and G parameters.


$$C_{i}={Block}_{C_{eq.1}}+\left({Block}_{C_{eq.1}}-{Block}_{i}\right)\times F+\frac{G}{\lambda }(1-F)$$ (9)

F is the Exponential term, G is the Generation rate, ${Block}_{C_{eq.1}}$ is the optimal solution set, and *Block*_*i*_ is the current block.

Evolutionary algorithms use the introduced function as a search space in solving benchmark problems. In the copy-move forgery detection problem, the problem space is the image. Blocks, which are introduced as output concentrations, are obtained by the SIFT function. The updated concentrations should be applied to the input image to complete the search result. To apply the new concentration obtained, Eqs (10) or (11) are used.

If the current concentration is a member of the solution population set, Eq (10) is applied to the image blocks. Applying Eq (10) makes the image blocks white. Eq (11) is applied if the current concentration is not part of the set of optimal solutions. In this case, dividing the standard deviation by 100 pushes the selected area to black.


$${Block}_{i}=STD(C_{i})\times {Block}_{i}$$ (10)

$${Block}_{i}=\frac{STD(C_{i})}{100}\times {Block}_{i}$$ (11)

In Eqs (10) and (11), STD is the standard deviation of the selected block. According to the range of the image in the range of 0 to 255, the maximum value of the standard deviation is two digits. Eq (10) increases the block values, and Eq (11) decreases the block values.

### 3.3. Merged neighbor blocks with Color Histogram Matching (CHM)

The EOM algorithm detects forged blocks but cannot expand the forgery region. More localization should be done on it to improve the detection accuracy. We use a color histogram matching technique to extract the required forgery regions of the image. Blocks adjacent to suspicious blocks are compared with each corresponding suspicious block using color similarity based on color histogram matching.

The color feature is computed using the color histogram for the eight neighboring blocks surrounding a suspected region block. The neighboring blocks are defined for each suspected region,${SR}_{i}=\left({MB}_{i}.\bar{{MB}_{i}}\right)$ as shown in Eq (12) [2]:

$${SR}_{ineighbor}=\left({MB}_{i\theta }.\bar{{MB}_{i\theta }}\right)$$ (12)

**Fig 5:**
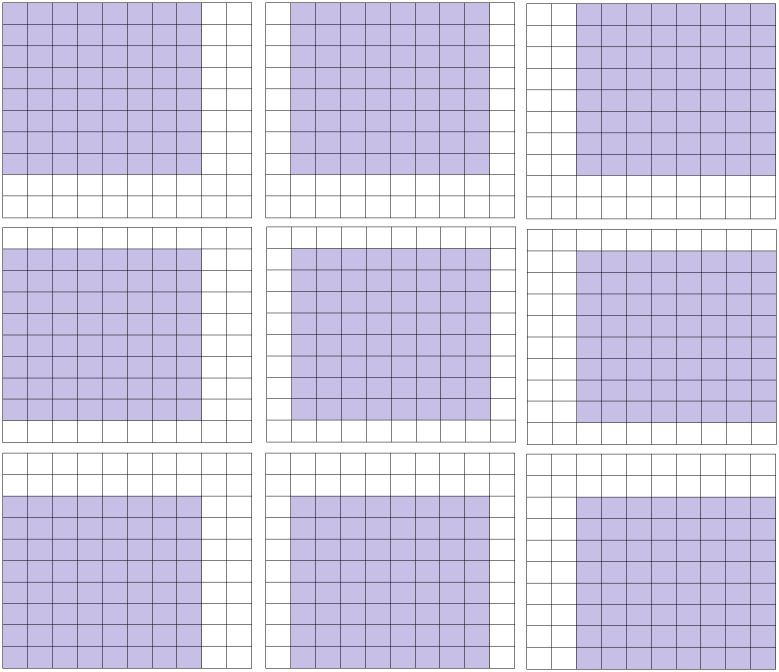
Neighborhood blocks with eight position.

Where ${MB}_{i}.\bar{{MB}_{i}}$ are the corresponding matching blocks and ${MB}_{i\theta }.\bar{{MB}_{i\theta }}$ are the neighbor blocks of ${MB}_{i}.\bar{{MB}_{i}}$ at an angle θ, where θ = 45, 90, 135, 180, 225, 270, 315, and 360 degrees. The system’s input is the blocks obtained with the help of the EOM algorithm (Fig 5). A color histogram is calculated for each adjacent block. Then, the Euclidean distance is calculated between the normalized color histograms of a suspect block and each of its eight adjacent blocks. Each block that has the most minor difference is selected for expansion. Any adjacent blocks may be selected as a suspicious blocks with the help of the SIFT method.

As a result, the suspicious block is expanded, and the detection is complete by expanding the forgery area.

After completing the forgery detection process, one must fill in the gaps. Morphological operations are used to fill the remaining gaps. The closing of the merged image f by a structuring element s is the process of a dilation followed by erosion, as shown in Eq (13) [2],

f⨀S=(f⊕S)⊖S (13)

Where ⊕ denotes dilation and ⊖ denotes erosion [2].

The dilation operation will cause the object boundaries to increase, and the subsequent erosion operation will partially reverse this expansion. Therefore, the concluding process serves to fill small openings and narrow gaps. A circle with a radius of 5 is employed as the structural component in the close operation. The preservation of the shape of the region is maintained as the close operation also fills in any gaps present.

The challenge at this stage is to optimally select blocks. We choose blocks with more forgery keypoints to effectively expand the fake area and achieve a more accurate result. Blocks with more keypoints obtained from SIFT are more likely to be expanded. For expansion purposes, erosion and dilation are utilized. The order of selection of blocks to apply functions, the number of keypoints will be fake.

**Fig 6:**
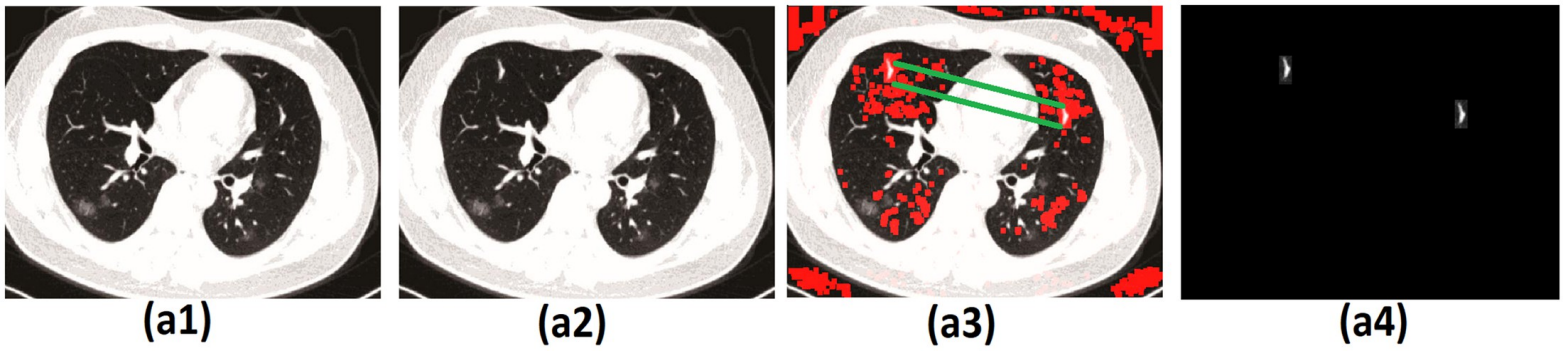
Image result of the proposed method (SEC), (a1) Main Image, (a2) Forged Image, (a3) Image Matching with SEC, (a4) Detection Result.

Fig 6 illustrates the outcome of the model that has been suggested. The model, as proposed, consists of three components, namely SIFT, EOM, and CHM. Image (a1) is the Main image, (a2) is the copy-move forgery image, (a3) is the blocks detected by SEC, and (a4) is the output image of the copy-move forgery area.

## 4. Results and discussions

### 4.1. Database

The proposed SEC method is applied to 300 medical images. This dataset, available on the website https://www.ctisus.com/teachingfiles, contains 100 original images, while the website https://github.com/DrAmiri/14020228.git contains 200 fake images.

Fake images are synthetically formed using image editing tools to study the performance of the developed method [18]. Results are given for simple forged, rotating forged (5, 10, and 15 degrees) and noisy forged images.

**Fig 7:**
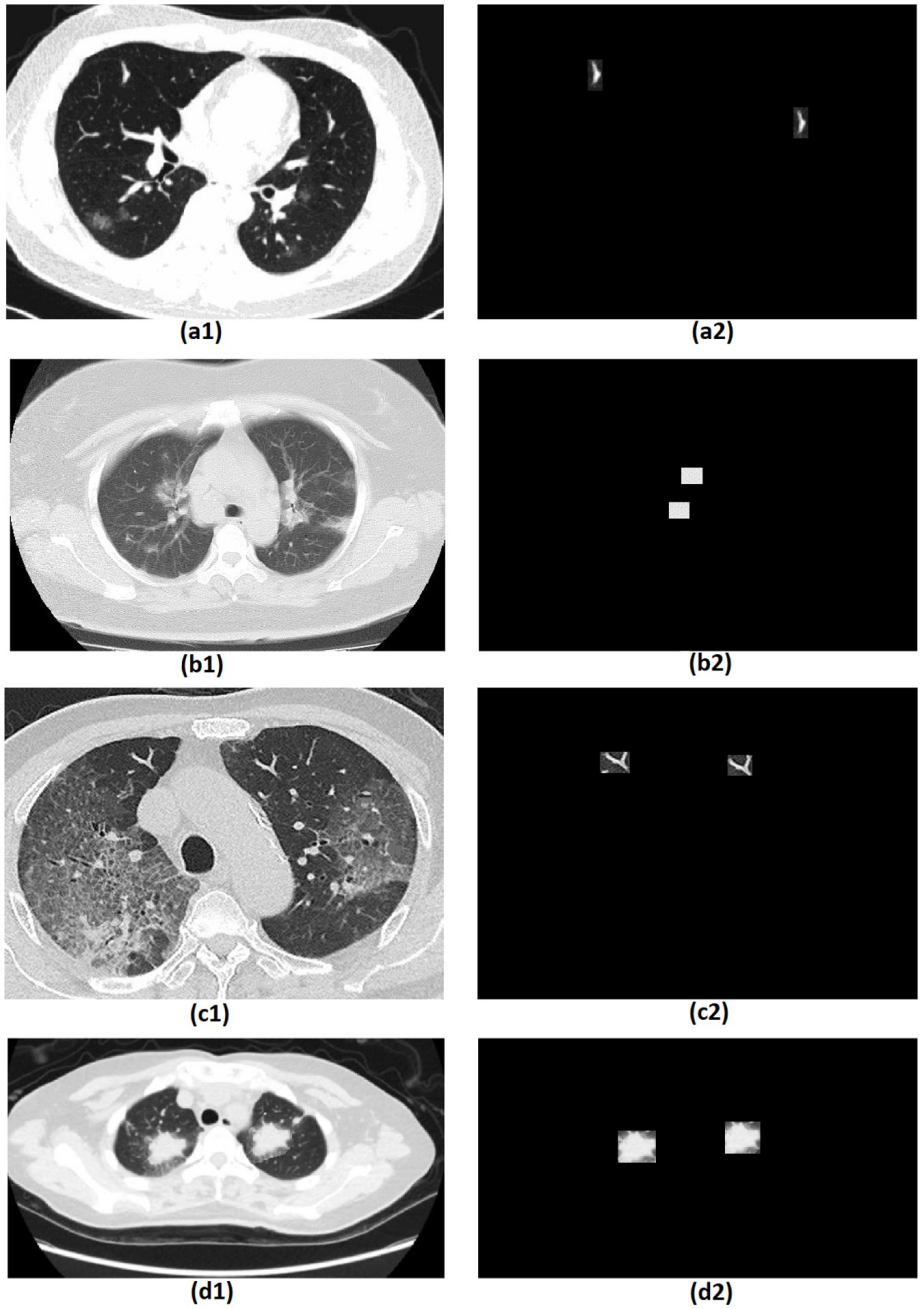
The proposed method in the copy-move forgery detection on the dataset. Main forged image and Detected forgery region.

Fig 7 shows applying the proposed model to the existing database. The images of the first group are fake, and the second group detected images. Based on the results, the proposed method can detect fake parts of the images to a large extent and return the appropriate blocks.

### 4.2. Performance measures

The image forging system aims to increase the accuracy of detecting and finding all pixels belonging to the tampered area. The function of forgery detection systems is tested on image level and pixel level. The function of forgery detecting areas at the image level is emphasized on whether an image is manipulated or not. In contrast, the forgery detection function at the pixel level focuses on the correct location of the manipulated areas.

Generally, three commonly used indexes, *precision* (Eq (14)), *recall* (Eq (15)), and *F*1 (Eq (16)), indicate the effectiveness of the method in discovering the image forging. They are calculated as [9]:

$$Sensitivity=\frac{A1}{A1+B1}$$ (14)

$$Specificity=\frac{A2}{A2+B2}$$ (15)

$$F1=2\times \frac{Precision.\;Recall}{Precision+Recall}$$ (16)

Where A1 is an image correctly detected as a forgery, B1 is an image wrongly labeled as original, A2 is correctly detected as original, and B2 is wrongly labeled as a forgery. The proportion of perfectly-recognized fake images among authentic fake images is what sensitivity means. The specificity metric shows the ratio of accurately identified genuine pictures in the original photos.

### 4.3. Comparison results and analysis

Various methods are utilized to examine some of the outcomes of image forgery detection in this section. The SEC algorithm is utilized to detect forgery in one of the algorithms that have been discussed. Although keypoint-based approaches possess the ability to detect counterfeit images automatically, their outcomes are imprecise. Other methods will be less powerful compared to the proposed algorithm if the image forgery detection has a higher accuracy.

Here, we provide the comparison outcomes of the SEC algorithm using various types and techniques of spoofing. The database consists of 300 images that are original, easily manipulated, rotated, and involve noise. The outcomes are presented in Table 1, divided into two sections: images without forgery and images with forgery. Images that have been manipulated are displayed in various forms such as basic forgery, rotation, and the addition of Gaussian noise.

**Table 1:** Comparison of the proposed method in the data set.

Actual class	Rate of Precision (%)	Rate of Recall (%)	Rate of F1 (%)	Specificity (%)	Sensitivity (%)
Original	100	100	100	100	99.82
Simple forgery images	100	95.6	97.75	99.02	97.36
Forgery images with rotation (5°)	94.8	94.9	94.84	92.10	89.86
Forgery images with rotation (10°)	90.7	91.1	90.89	89.11	86.79
Forgery images with rotation (15°)	90.1	90.5	90.29	88.33	82.56
Forgery images with noise	93.6	89.0	91.24	91.66	89.43
Average	94.86	93.51	94.16	93.37	90.97

Table 1 shows the performance differences in the types of fake images in the database. The precision of detecting non-fake images is excellent. In contrast, it detects fake images with about 94%, which is a very good result.

**Fig 8:**
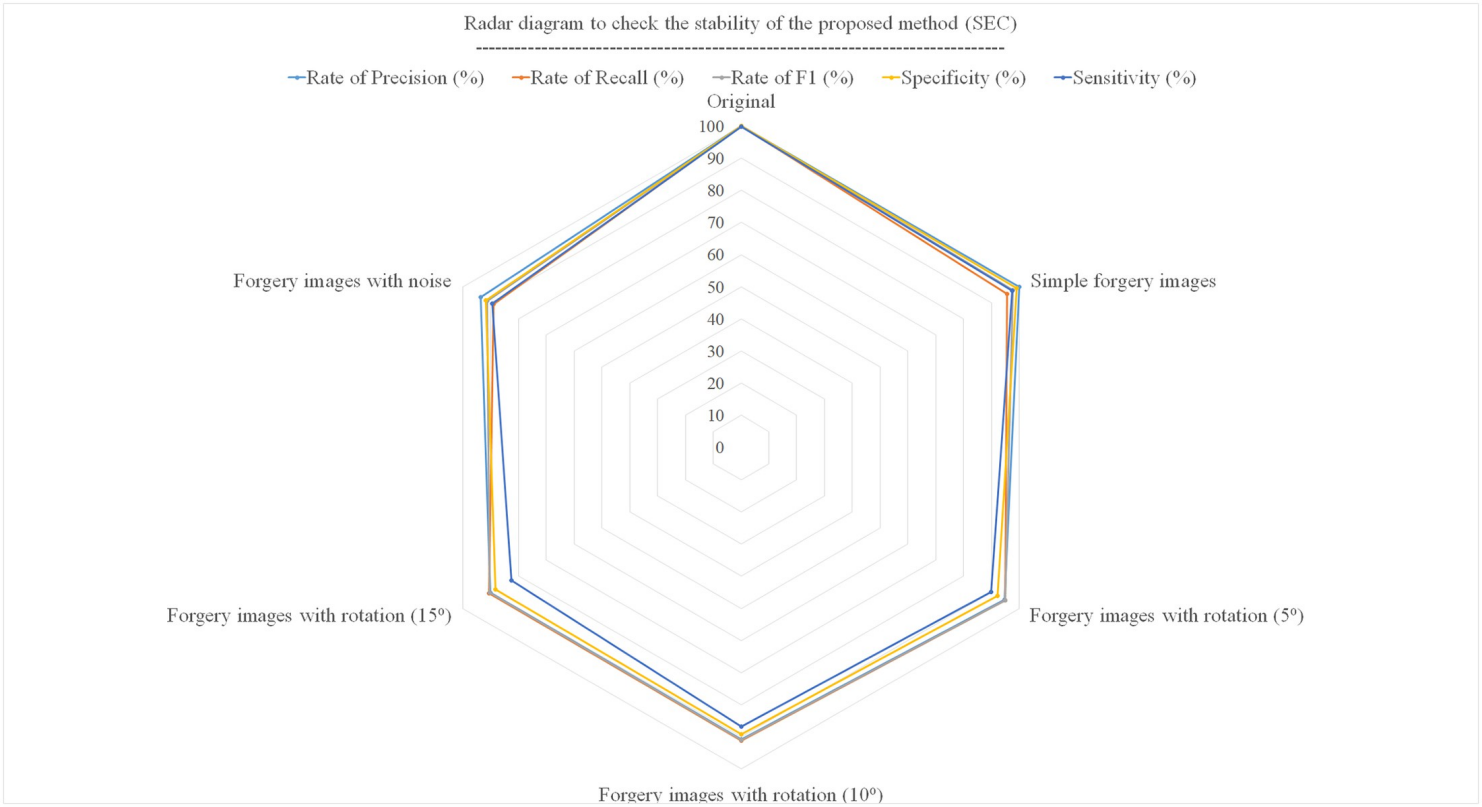
Radar diagram to compare the stability of the proposed method (SEC) in medical images forgery.

The radar diagram in Fig 8 shows the stability of forgery detection for different modes. In rotated images, the accuracy is lower as the number of rotations increases. The existing noise images were created using a Gaussian function with a factor of 0.1. The proposed method’s image forgery detection accuracy in Gaussian noise images is about 90%.

To check the power performance of the proposed method, should make a performance comparison with other methods.

Table 2 shows the high-performance precision of the proposed method on the existing database. In addition to Precision, there is excellence in the Recall and F1 criteria. The results in Table 2 show that the introduced method (SEC) has the highest F1 (98.93%), followed by 98.47% in CMFDEOA, and 96.91% in SMGM for the forged images.

**Table 2:** Comparison of the proposed method and other methods.

Algorithms		Rate of Precision (%)	Rate of Recall (%)	Rate of F1 (%)
SMGM [18]	Original Image	100	100	100
	Forged Image	100	94.0	96.91
CSM [18]	Original Image	100	100	100
	Forged Image	100	82.0	90.11
BAM [35]	Original Image	100	99.0	99.49
	Forged Image	86.5	90.5	88.45
MROGH [45]	Original Image	-	-	-
	Forged Image	93.6	91.7	92.6
Gabor Filter [46]	Original Image	-	-	-
	Forged Image	83.3	90.9	86.92
SIFT-ACO [47]	Original Image	-	-	-
	Forged Image	90.0	89.63	89.81
CMFDEOA [48]	Original Image	100	100	100
	Forged Image	100	97.0	98.47
Proposed Method (SEC)	Original Image	100	100	100
	Forged Image	100	97.9	98.93

The results in Table 2 show an improvement of about 2%. Therefore, the proposed method has improved the results in forgery detecting areas. The most important feature of this method is the selection of optimal blocks that other methods have not been able to detect.

**Fig 9:**
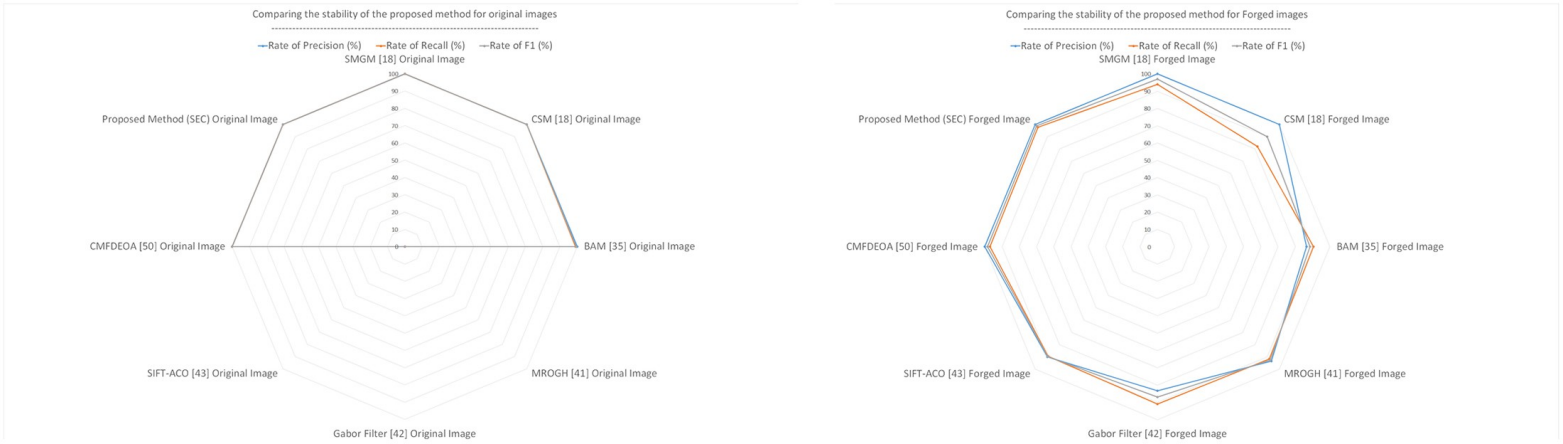
Radar diagram to compare the stability of the proposed method (SEC) with other methods.

Fig 9 shows that the proposed method has obtained better results than other methods in all three-evaluation criteria.

We compare our method “SEC” with other methods: SMGM [18], CSM [18], density-based clustering [49], and LPG [50]. The results η⃗ on five runs over the test set are presented in Table 3. It is easily observed that our proposed SEC smashes all the other four comparison baseline methods in all indicators.

**Table 3:** Comparison to State-of-the-art approaches (%).

Approach	*η*1	*η*2	*η*3	*η*4	*η*5
SMGM [18]	93.01	93.60	95.12	94.50	94.20
CSM [18]	82.81	84.10	81.11	81.20	81.61
density-based clustering [49]	94.21	94.19	94.01	93.18	91.44
LPG [50]	92.01	92.08	91.97	91.89	91.87
SEC (Ours)	97.70	97.12	98.24	97.80	98.23

The reason why our method is better than other methods is three-fold: (i) We choose the Equilibrium optimization algorithm, and (ii) We used a combination of block and key point methods.

Most forgery detection methods determine the authenticity of images using keypoint or block techniques. Each of them has its own issues, but the proposed method has successfully addressed multiple problems by merging two forgery detection models. Also, due to the use of the equilibrium optimization algorithm, it has been able to optimize the diagnosis process. The issue of expanding the area of forgery is another issue that has been addressed in a small number of articles. The proposed method has enabled the expansion of the forgery area through the use of morphological functions. Since deep learning methods struggle at the pixel level, further research into the proposed method may lead to improved results in expanding the detection area in rotated, noisy, and multiple images.

## 5. Conclusion

Forging an image is a common way to evade the law. In the medical industry, it is tough to detect fake images because image processing tools and software are potent. Block-based or key-based methods can detect medical image forgery. The method introduced is an EOA-based algorithm and SIFT-based called SEC, which focuses on detecting copy-move fraud. Experimental analysis of the proposed method showed its effectiveness in copy-move forgery detection for medical images. This method has obtained a higher accuracy than other investigated methods. Tables 1 and 2 show the accuracy of the proposed method, which is better than different algorithms. Tables 1 and 2 also show that the proposed copy-move forgery method obtains forgery points with 98.93% in F1 for the medical image dataset. In the future, we will try to improve the performance of the proposed method, which can better expand the adjacent areas of forged blocks, and as a result, expand the forgery area well. However, with the advancement of digital systems, the proposed model should also be tested in online models. The identification of counterfeit images, videos, and voices has now captured the interest of researchers. Also In future research, forgery detection in images could be explored and evaluated using deep neural networks.
